# Patterns of spinal cord atrophy in HTLV-1 associated myelopathy/ tropical spastic paraparesis (HAM/TSP)

**DOI:** 10.1186/1742-4690-12-S1-P32

**Published:** 2015-08-28

**Authors:** Emily Charlip, Winston Liu, Raya Massoud, Jeanne Billioux, Breanna Caruso, Joan Ohayon, Govind Nair, Steven Jacobson

**Affiliations:** 1 Neuroimmunology Branch, National Institute of Neurological Disorders and Stroke, NIH, Bethesda, MD, USA; 2 Fischell Department of Bioengineering, University of Maryland, College Park, MD, USA

## 

Spinal cord inflammation and atrophy contribute to debilitating symptoms in HAM/TSP. We have developed a robust and fast algorithm to determine average cross-sectional area in cervical (c-spine) and thoracic (t-spine) spinal cords by tracing contours perpendicular to the edge in T1-weighted MRI images. The cross-sectional areas in the c- and t-spines were determined in 25 HAM/TSP, 10 asymptomatic carriers (AC) and 10 healthy volunteer (HV) subjects. To date, we have followed 8 of the HAM/TSP patients longitudinally over a two-year period. When compared to the HV data, the HAM/TSP spinal cord profiles fell into four general categories: atrophic entire spine (48%), atrophic t-spine (32%), atrophic c-spine (8%), and normal (12%). The majority of ACs had similar spinal cord profiles to those in the HV group, however, 3 ACs showed a pattern similar to HAM/TSP. As a group, both HAM/TSP c- and t-spines were significantly lower than those of HV (p<0.01). In the 8 patients with follow-up scans, spinal cord size showed an overall decreasing trend over time. In a rapidly progressing patient with the shortest disease duration, we could estimate spinal cord atrophy at a rate of 11% a year in the thoracic cord. In addition, change in proviral load negatively correlated with change in both c- and t-spine cross-sectional area (p<0.05) for patients with shorter disease duration and increasing proviral loads (i.e. an increase in proviral load was associated with a more atrophic cord). These results suggest that the pattern of spinal cord tissue damage is specific to the underlying inflammatory disease, a finding that has direct implications for the use of average cross-sectional spinal cord area as a surrogate end point for clinical trials.

